# The Effectiveness of Transcranial Brain Stimulation in Improving Clinical Signs of Hyperkinetic Movement Disorders

**DOI:** 10.3389/fnins.2015.00486

**Published:** 2016-01-07

**Authors:** Ignacio Obeso, Antonio Cerasa, Aldo Quattrone

**Affiliations:** ^1^Centro Integral en Neurociencias A. C. (CINAC), HM Hospitales – Puerta del Sur. MóstolesMadrid, Spain; ^2^Center for Networked Biomedical Research on Neurodegenerative DiseasesMadrid, Spain; ^3^Neuroimaging Research Unit, Institute of Molecular Bioimaging and Physiology - National Research CouncilGermaneto, Italy; ^4^Neurology Unit, Institute of Neurology, University “Magna Graecia”Catanzaro, Italy

**Keywords:** rTMS, Parkinson's disease, levodopa-induced dyskinesias, essential tremor, dystonia

## Abstract

Repetitive transcranial magnetic stimulation (rTMS) is a safe and painless method for stimulating cortical neurons. In neurological realm, rTMS has prevalently been applied to understand pathophysiological mechanisms underlying movement disorders. However, this tool has also the potential to be translated into a clinically applicable therapeutic use. Several available studies supported this hypothesis, but differences in protocols, clinical enrollment, and variability of rTMS effects across individuals complicate better understanding of efficient clinical protocols. The aim of this present review is to discuss to what extent the evidence provided by the therapeutic use of rTMS may be generalized. In particular, we attempted to define optimal cortical regions and stimulation protocols that have been demonstrated to maximize the effectiveness seen in the actual literature for the three most prevalent hyperkinetic movement disorders: Parkinson's disease (PD) with levodopa-induced dyskinesias (LIDs), essential tremor (ET) and dystonia. A total of 28 rTMS studies met our search criteria. Despite clinical and methodological differences, overall these studies demonstrated that therapeutic applications of rTMS to “normalize” pathologically decreased or increased levels of cortical activity have given moderate progress in patient's quality of life. Moreover, the present literature suggests that altered pathophysiology in hyperkinetic movement disorders establishes motor, premotor or cerebellar structures as candidate regions to reset cortico-subcortical pathways back to normal. Although rTMS has the potential to become a powerful tool for ameliorating the clinical outcome of hyperkinetic neurological patients, until now there is not a clear consensus on optimal protocols for these motor disorders. Well-controlled multicenter randomized clinical trials with high numbers of patients are urgently required.

## Introduction

Alteration in dynamics of neural connectivity is the hallmark of motor and behavioral disease in humans. Brain connectivity affected by functional deficits will either produce exacerbated or reduced brain signal and thus the observed clinical symptomatology. In the motor domain, presence of hyperkinetic movement disorders is typically manifested as increased muscular activity that leads to involuntary and unwanted movements, abnormal postures or combination of both. These are present in several neurological disorders, such as essential tremor (ET), dystonia, and Parkinson's disease (PD). In contrast, hypokinetic movement disorders represent loss of vigor and movement that produces rigidity and the inability to initiate and terminate actions efficiently, present in bradykinesia or freezing of gait in PD. Current treatments are mainly pharmacological, but recently functional surgery has made progress in remediation of uncontrolled and unwanted motor disorders (Fasano and Lozano, 2015).

The basal ganglia are considered the main neurodegenerative site of hyper- and hypo-kinetic movement (Middleton and Strick, 2000; Hamani et al., 2004). Due to its strict relationship with several brain regions, the basal ganglia are considered the principal hub of the neural pathways involved in motor control, which included other regions such as the subthalamic nucleus (STN), globus pallidum (GP), thalamus, together with the supplementary motor area (SMA), motor cortex (M1), and frontal regions (Alexander et al., 1986; Kehagia et al., 2013). In the last few years, advances in the neurophysiological and neuroimaging fields have provided alternative scenarios for understanding the neurobiological mechanisms of motor disorders. Indeed, several lines of evidence support the notion that others structures, outside traditional striato-thalamo-cortical pathways, are strongly involved. In particular, the cerebello-thalamic circuitry (Pinto et al., 2003; Lehéricy et al., 2013) as well as intra-cortical connections between the premotor cortex and the inferior frontal cortex (IFC), would seem to play a key role in the dysfunctional pathophysiological model of some hyperkinetic motor disorders (Herz et al., 2014; Cerasa et al., 2015).

When traditional treatments fail or do not reach the expected motor benefit, it is now possible to modulate the pathological level of cortical activity using invasive methods such as deep brain stimulation (Diamond and Jankovic, 2005). However, considerable effort is being made on applying other methods that are non-invasive, less costly, and capable of producing beneficial effects in the long-term. The increasing number of research and clinical protocols using non-invasive brain stimulation protocols in patients with neurological conditions show intermixed effects and reports. To date, therapeutic trials using repetitive transcranial magnetic stimulation (rTMS) in PD, ET or dystonia have reported some controversial findings. The use of inhibitory brain stimulation to reduce excessive and abnormal cortical activity in hyperkinetic motor disorders is a potential tool to remediate motor control, posture, muscle tone, and cognitive problems, but considerable effort is needed to test the multiple available protocols when using brain stimulation tools in neurological patients (Ridding and Rothwell, 2007; Elahi et al., 2009).

The present review aims to focus on studies using transcranial brain stimulation protocols to modulate hyperkinetic neurological disorders aimed at clarifying the optimal conditions in which non-invasive stimulation may be used in movement disorders. We selected studies with constrained search in PubMed and Medline using as search terms: dyskinesias, dystonia, and ET in combination with widely used brain stimulation terms: TMS, rTMS, and TBS, from inception to September 25, 2015. Publication lists of relevant studies were later scanned for potential eligible articles. We summarize key technical aspects of rTMS with effective results for PD, ET, and dystonia to propose focused research plans to increase the positive impact of non-invasive brain stimulation in clinical practice.

### rTMS protocol for therapeutic purpose

rTMS has effects on the brain and behavior that outlast the period of stimulation due to plastic changes of long-term potentiation or depression in synaptic connections amongst cortical networks. Regions or networks with suboptimal functioning after brain damage or neurodegenerative disease are potential candidates for neuromodulation therapy. So far, the therapeutic use of rTMS has been proved effective in patients with major depression refractory to regular treatment (George et al., 2013). In neurological realm, movement disorders has received much attention with regard to rTMS therapeutic studies. However, experiments in healthy subjects suggest that rTMS protocols have short-lived after-effects. Hence, clinical neuroscience encounters a challenge with aim boosting longer time-periods of beneficial effects in patient's quality of life.

Despite illness, several rTMS protocols may be used for therapeutic purpose (for review see Ridding and Rothwell, 2007). The key aspect to consider is how to prolong rTMS positive effects in clinical conditions and quality of life. Current rTMS protocols apply *low frequency* (<1 Hz) or *high frequency* (>1 Hz), as well as *single* rTMS or *multiple* rTMS sessions. Generally, high frequency stimulation induces an increase in cortical excitability and low frequency stimulation causes a decrease in cortical excitability. To benefit plastic and long-term rTMS potentiation, multiple sessions tend to show stronger and cumulative effects in clinical and behavioral measures. An alternative use of rTMS is theta burst stimulation (TBS), consisting of short, repeated bursts of TMS pulses at 50 Hz (Huang et al., 2005). Again, the use of TBS allows decrease (using continuous TBS) or increase (using intermittent TBS) of cortical excitability using different sets of magnetic trains.

The fact that cortical baseline activity may be either hyperexcitable or hypoexcitable has formed the idea of using low-frequency rTMS to treat disorders with marked cortical hyperexcitability, while using high frequency rTMS in conditions with low cortical excitability. For this reason, in hyperkinetic motor disorders the rationale behind the application of rTMS protocol is to reduce abnormal cortical hyperexcitability, although this is not true in all the circumstances as it depends on several methodological and clinical factors, discussed in the present review.

## Therapeutic potential of rTMS in parkinson's disease patients

PD is primarily a disorder of response initiation characterized by an excessive motor inhibition. In particular, bradykinesia (slowness of voluntary movements), tremor, rigidity, and gait problems are cardinal motor signs in PD, greatly improved by treatments with dopamine replacement therapy. However, PD-related neurochemical changes are long-lasting and difficult to contrast by pharmacological interventions. For this reason, new treatment strategies have been proposed. rTMS has been studied as an intervention to ameliorate motor symptoms (Edwards et al., 2008; Elahi et al., 2009), including rigidity and bradykinesia, motor complications of therapy (e.g., dyskinesias) and non-motor symptoms, mainly depression and speech (Lefaucheur et al., 2004). Despite a large heterogeneity among these studies (Koch, 2013), it was proposed that high frequency rTMS (i.e., 5 Hz) applied over M1 could turn as a gold-standard use in PD to significantly reduce motor signs as measured by UPDRS-III (Elahi et al., 2009). Moreover, the diverse results provided by the literature indicate updating in future interventions, which will necessitate separation of PD motor signs in an attempt to separate the diverse pathophysiology present in tremor, bradykinesia, rigidity, and gait problems. In case where such separation turns successful, perhaps we could foresee new ways of understanding and treating PD symptoms alternatively.

### rTMS in parkinson's disease patients with levodopa-induced dyskinesias

Nowadays, treating secondary motor signs related to PD treatments is a possibility based on clear pathophysiological models to reach effective targets. Despite pharmacological or non-pharmacological interventions, after 4–6 years of levodopa therapy, a significant proportion of patients exhibit a decline in the therapeutic efficacy of levodopa and develop disabling motor symptoms, termed levodopa-induced dyskinesias (LIDs). The time-to-onset and severity of this motor complication show large individual variability thus limiting the long-term use of levodopa and clinical strategies aimed at reducing LIDs manifestation.

In the last few years, a considerable effort has been made to understand the neurobiological basis of this motor complication. LIDs are classically ascribed to the degree of nigrostriatal neurodegeneration and striatal changes associated with chronic levodopa therapy (Obeso et al., 2000). These interact to induce maladaptive striatal plasticity, which has the effect of altering neuronal activity in striato-pallidal circuits. The pioneering works of Rascol et al. (1998) and Brooks et al. (2000) demonstrated *in vivo* that these abnormal neuronal firing patterns extended on the brain cortex mainly including the sensorimotor areas of the cortico-basal ganglia loop.

After these first functional neuroimaging studies, for a long time no additional neuroimaging investigations have been performed on LIDs patients. From 2010 to date, new functional and structural neuroimaging studies have shed new light on the pathophysiological mechanisms underlying LIDs suggesting that that LIDs-related symptoms may originate in brain network beyond the “classical” basal ganglia dysfunctional model, including cortical regions strongly involved in motor inhibition processes. Indeed, what has clearly been demonstrated was that PD patients with LIDs are characterized by dysfunctional coupling between the prefrontal cortex, including the right IFC and the SMA and basal ganglia measured at rest (Cerasa et al., 2015), during a simple finger-tapping task (Cerasa et al., 2012) or during a GoNo-Go task (Herz et al., 2014, 2015). Moreover, these functional abnormalities in LIDs patients were also mirrored by abnormal anatomical changes detected in the SMA and IFC (Cerasa et al., 2011, 2013a,b). These findings have already raised an interesting scientific debate on the toxic effects of levodopa on brain morphometry (Vernon and Modo, 2012; Cerasa et al., 2014) and on the hypothetical role of the prefrontal cortex as a new target for brain stimulation useful to decrease the severity of LIDs (Cerasa and Quattrone, 2014a,b; Obeso and Strafella, 2014a,b; Rothwell and Obeso, 2015), seen to improve motor inhibition due to compensatory processes of interconnected regions (Obeso et al., 2013; Zandbelt et al., 2013).

Indeed, to treat secondary effects of principal treatments in PD such as LIDs is also an actual necessity and priority. Guided by imaging results, rTMS over regions showing functional overactivity in LIDs was reported either over the SMA (Koch et al., 2005; Brusa et al., 2006) or over the IFC (Cerasa et al., 2015) (Table 1). Otherwise, no significant or moderate effects emerged when TMS protocol was applied over the primary motor cortex (Wagle-Shukla et al., 2007; Filipovic et al., 2009; Kodama et al., 2011; Filipović et al., 2013; Cerasa et al., 2015). In particular, the Koch's group was the first in using rTMS approach with therapeutical purpose (Koch et al., 2005). In 2005, they demonstrated that one single session of rTMS at low frequency (1 Hz) over the SMA produced significant motor improvements in eight patients with LIDs. The rationale behind the choice to stimulate SMA is based either on previous neuroimaging findings describing functional overactivity in this region (Rascol et al., 1998; Brooks et al., 2000) or on the notion that repeated sessions of premotor cortex stimulation induces cumulative changes in the excitability over the primary motor cortex (Bäumer et al., 2003). With this in mind, Brusa et al. (2006) tried to translate this single TMS protocol in a prolonged therapeutic session (5 days), failing to demonstrate a clear beneficial effect. Contrarily, prolonged session (2 weeks) applied on the bilateral cerebellar cortex using high frequency (50 Hz) cTBS, showed persistent clinical beneficial effects in LIDs patients for up to 4 weeks (Koch et al., 2009). To explain this discrepancy, these authors proposed that this might be dependent upon the fact that the cerebellum has greater plastic mechanisms involved in motor learning (Ito, 2008) compared to SMA and therefore could be susceptible to more sustained rTMS-induced changes, thus leading to marked clinical beneficial effects. Moreover, recent evidence suggested a causal role of the effective cerebello-cortical connectivity in motor inhibition (Picazio and Koch, 2015), a cognitive domain strongly involved in the pathophysiological mechanisms of LIDs (Cerasa et al., 2015). The intimate link between motor inhibition and LIDs has also been confirmed in a recent study (Cerasa et al., 2015) where it was demonstrated that a single session of continuous but not intermittent or sham TBS applied over the right IFC was able to significantly reduce the amount of dyskinesias as measured by the conventional abnormal involuntary movement scale (AIMS).

**Table 1:** **rTMS application on PD with LIDs**.

**References**	**Sample**	**TMS protocol**	**Anatomical localization**	**Main findings**
Koch et al., 2005	8 Dyskinetic PD	Single Session rTMS train at 1 Hz or 5 Hz	SMA	Single Session Low frequency (1 Hz): reduced AIMS after 15 min
				Single Session High frequency (5 Hz): induced a slight but not significant effect
Brusa et al., 2006	10 Dyskinetic PD	Single and Prolonged (5 days) sessions rTMS train at 1 Hz	SMA	Single Session Low frequency (1 Hz): reduced AIMS and improved UPDRS scores after 15 min
				Prolonged Session Low frequency (1 Hz): failed to enhance beneficial effects
Wagle-Shukla et al., 2007	6 Dyskinetic PD	Prolonged (2 weeks) sessions rTMS train at 1 Hz	M1	Prolonged Session Low frequency (1 Hz): induced a slight but not significant effect
Filipovic et al., 2009	10 Dyskinetic PD	Prolonged (4 days) sessions rTMS train at 1 Hz	M1	Prolonged Session Low frequency (1 Hz): induced a modest beneficial effect
Kodama et al., 2011	Case Report PD with painful off-period dystonia	Single Session rTMS train at 0.9 Hz	M1	Single Session Low frequency over M1: reduced painful dystonia and walking disturbances
			SMA	Single Session Low frequency over SMA: induced no significant effects
Filipović et al., 2013	Case Report PD with diphasic dyskinesia	Prolonged (4 days) sessions rTMS train at 1 Hz	M1	Prolonged Session Low frequency (1 Hz): yielded beneficial effects in the upper limb
Koch et al., 2009	10 Dyskinetic PD	Prolonged (2 weeks) sessions cTBS 3 pulse bursts at 50 Hz	Cerebellum	Prolonged Session High frequency (50 Hz): yielded beneficial effects
Cerasa et al., 2015	11 Dyskinetic PD	Single Session cTBS 3 pulse bursts at 50 Hz	Right Inferior Frontal Cortex	Single Session High frequency (50 Hz): reduced AIMS after 45 min
			M1	Single Session High frequency (50 Hz): failed to enhance beneficial effects

PD, Parkinson's disease; LIDs, Levodopa-Induced Dyskinesias; SMA, Supplementary Motor Area; M1, Primary motor cortex; rTMS, repetitive Transcranial Magnetic Stimulation; iTBS, intermittent theta burst Transcranial Magnetic Stimulation; cTBS, continuous theta burst Transcranial Magnetic Stimulation; AIMS, Abnormal Involuntary Movement Scale.

The primary goal of the motor inhibition system (mainly composed by STN, basal ganglia, SMA, and IFC) is to control/modulate the primary motor output pathway. Idiopathic PD is primarily a disorder of response initiation characterized by an excessive motor inhibition (i.e., akinesia, bradykinesia), whereas LIDs are clearly a clinical expression of disinhibition of movement. For this reason, the recent neuroimaging evidence strongly supports the idea that dysfunctions of the primary motor system in LIDs patients are related to that of motor inhibition pathway. However, it remains to be clarified why clinical beneficial effects are evident after rTMS over the cortical regions involved in the motor inhibition system (SMA and IFC), whereas brain stimulation on the primary motor cortex produced conflicting results (Wagle-Shukla et al., 2007). Indeed, Wagle-Shukla et al. (2007), using a prolonged session (2 weeks) of low frequency (1 HZ) rTMS over the primary motor cortex, did not report evident clinical improvements in 6 PD patients with LIDs. This preliminary evidence has also been confirmed in a recent study (Cerasa et al., 2015), despite the employment of a different TMS protocol [single session high-frequency (50 Hz) cTBS]. Three additional studies, otherwise, reported moderate evidence about the role of the primary motor cortex as potential stimulation site for LID treatment. First, Filipovic et al. (2009), using low-frequency rTMS (1 Hz) for 4 consecutive days in 10 PD patients with LIDs, reported residual beneficial clinical effects in dyskinesia severity. With the same TMS protocol, these authors found an increased beneficial effect also in one PD patient with diphasic dyskinesia, which is far less studied than more common peak-of-dose dyskinesias (Filipović et al., 2013). Finally, in another case report, 0.9 Hz rTMS over primary motor area significantly reduced the painful dystonia and walking disturbances in one dyskinetic patient with painful off-period dystonia (Kodama et al., 2011).

To sum up, the current literature on therapeutic trials of rTMS in PD patients with LIDs is in its relative infancy, and nowadays there is insufficient information to support evidence-based clinical protocols. However, the search for the most effective protocol leads us to the conclusion that brain stimulation on cortical regions part of the motor inhibition network (IFC, SMA, and cerebellum) might be highly promising as therapeutical sites for treatment of LID. Otherwise, evidence provided by rTMS over the primary motor cortex requires further confirmation. Indeed, while in idiopathic PD a plethora of studies demonstrated the beneficial effects on motor symptoms after high-frequency stimulation of the primary motor cortex (Edwards et al., 2008), in dyskinetic patients the high clinical heterogeneity, as well as variability in TMS protocols prevents us from making a general conclusion about these findings. The lack of consistency is also dependent upon the fact that advanced neuroimaging has not yet clarified how levodopa influences neurofunctional activity in the motor cortex.

## The potential use of rTMS to treat dystonia

Dystonia is a hyperkinetic movement disorder mainly characterized by excessive and painful muscle contraction producing muscle twists, abnormal posture, and inefficient moves. Body limbs involved in such muscles alteration classify the diverse types of dystonia. Focal dystonia are those where abnormal participation of muscles and gestures give raise to painful postures within an isolated body region. Meanwhile, segmental dystonia must involve two or more adjacent body regions and generalized dystonia, which affects upper and lower limbs of the body (Marsden, 1976). According to its etiology, dystonia can be divided into primary dystonia, dystonia plus syndrome or secondary dystonia (Marsden, 1976). Primary dystonia corresponds to those patients showing no brain lesions as revealed by structural MRI scans. It is well known that primary dystonia can be task-specific, altering movements involved in fine motor control (such as writer's cramp), speaking (dysphonia), playing piano, or running (Breakefield et al., 2008). This dystonia form may be idiopathic or genetic, based on a variety of more than 30 genes involved in the disease (Bragg et al., 2011). Secondary dystonia results from stroke or traumatic brain injury or induced by certain treatments thus has a certain origin. However, the causes of most dystonia are unknown but some monogenic subtype alterations (in DYT1, DYT6, or DYT13) are considered potentially relevant in developing dystonic motor symptoms (Bragg et al., 2011).

Considering the etiology heterogeneity in dystonia, its pathophysiological model may vary across dystonia subtypes. Based on clinico-pathological studies in patients with symptomatic dystonia (Marsden et al., 1985) and intracranial recordings from the GPi and thalamus (Vitek et al., 1999; Zhuang et al., 2004), dystonia is considered a basal ganglia disorder (Berardelli et al., 1998; Zheng et al., 2012). Indeed, DBS produces a significant positive response over the GPi (Vidailhet et al., 2005) and reduces metabolic activity over important cortical regions part of fronto-striatal loops [i.e., the dorsolateral prefrontal cortex (DLPFC) or the orbitofrontal cortex (OFC); Detante et al., 2004]. Recent findings from neuropathological data show in a large cohort of adult and child dystonia significant reductions of substantia nigra neurons as compared to controls (Iacono et al., 2015). This evidence has also been confirmed by positron emission tomography (PET) studies. Indeed, using dopaminergic markers at rest, some groups have pinpointed cell loss over striatal and cortical regions in primary dystonia (Otsuka et al., 1992; Berman et al., 2013). Moreover, increased glucose metabolism over the lentiform nucleus and cortical motor regions including SMA, lateral premotor cortex, anterior cingulate cortex (ACC), and DLPFC have also been reported in primary dystonia (Eidelberg et al., 1995; Odergren et al., 1998; Ibáñez et al., 1999; Pujol et al., 2000; Oga et al., 2002; Butterworth et al., 2003; Lerner et al., 2004). Functional alterations in dystonic patients were also coupled by underlying anatomical brain abnormalities. Indeed, patients with cervical dystonia, blepharospasm, or writer's cramp are characterized by anatomical changes in the basal ganglia, motor and premotor cortices, cerebellum and SMA (Eidelberg et al., 1995; Berardelli et al., 1998; Draganski et al., 2003; Zheng et al., 2012).

However, dystonia is not only considered to be dependent upon the basal ganglia-thalamo-cortical pathway (Breakefield et al., 2008), but recent evidence strongly highlights the involvement of the cerebellar cortex and its direct connections with the motor cortex (Lehéricy et al., 2013; Neumann et al., 2015). Cerebellar modulation over motor cortex seems to be compromised in dystonia patients and M1 excitability (i.e., intra-cortical facilitation) seems responsive to cerebellar rTMS (Brighina et al., 2009). However, it should bear in mind that although dystonic patients are not characterized by evident cerebellar motor signs (i.e., loss of balance or frequent falling), it has been proposed that the cerebellum in dystonia patients might be involved in compensatory modulation of the abnormal activity detected in the motor cortex, or as a potential effective input to modulate basal ganglia dysfunctional state (Wu and Hallett, 2013). Moreover, previous evidence points to altered cerebellar activation along the inhibitory motor circuits in dystonia (Huang et al., 2011; Koch et al., 2014), thus increasing the probability of such loops as potential candidate for neuromodulation.

The role of cortico-striatal and cerebellar-thalamo-cortical loops in dystonia, thus support two open accesses to cortical neuromodulation over motor, premotor, or cerebellar targets. The target location problem in dystonia seems rather straightforward based on current pathophysiological knowledge. So far, studies using rTMS to treat dystonia motor signs have reported beneficial clinical effects when targeting stimulation to motor (Odergren et al., 1998; Ibáñez et al., 1999; Pujol et al., 2000; Oga et al., 2002; Butterworth et al., 2003; Lerner et al., 2004; Murase et al., 2005; Allam et al., 2007; Angelakis et al., 2013; Berman et al., 2013) or somatosensory regions (Borich et al., 2009; Havrankova et al., 2010), but less clinical beneficial effects after cerebellar stimulation (Koch et al., 2014; Sadnicka et al., 2014) (see Table 2). Positive and acute effects after cerebellar stimulation in one study (Koch et al., 2014) offer new insights to further assess stimulation protocols with aim maintenance of prolonged positive effects (although not every study assessed long-term effects, Table 2). However, the gold-standard in dystonia seems to be targeting motor regions that produce functional changes over basal ganglia (Bharath et al., 2015).

**Table 2:** **rTMS application on dystonia**.

**References**	**Sample**	**TMS Protocol**	**Anatomical localization**	**Main findings**
Siebner et al., 1999	16 WC	Single session rTMS at 1 Hz, placebo controlled	M1	Single session yielded positive results as measured by pen pressure reductions and self-reported improvement
Lefaucheur et al., 2004	3 secondary dystonia	Prolonged sessions (5 consecutive days) rTMS at 1 Hz	Premotor	Prolonged session yielded positive results in movement rating scale and decrease in painful axial spams
Murase et al., 2005	9 WC	Single session (1 day) rTMS at 0.2 Hz	Premotor	Single session yielded positive results over premotor site, in decrease contraction and pen pressure
			SMA	
			M1	
Tyvaert et al., 2006	8 WC	Single session (1 day) rTMS at 1 Hz	Premotor	Single session yielded positive results in handwriting velocity and decreased discomfort
Allam et al., 2007	1 cervical dyst./WC	Prolonged sessions (5 consecutive days) rTMS at 1 Hz	Premotor	Prolonged session yielded positive results in a single case study in cervical dystonia
Borich et al., 2009	6 FHD 9 HC	Prolonged sessions (5 consecutive days) rTMS at 1 Hz	Premotor	Prolonged session rTMS yielded reduced cortical excitability and improved handwriting performance were observed and maintained at least 10 days
Havrankova et al., 2010	20 WC	Prolonged sessions (5 consecutive days) rTMS at 1 Hz	Somatosensory	Prolonged sessions yielded positive results in subjective and objective writing maintained for 3-week time period
Schneider et al., 2010	5 WC	Single session (1 day) rTMS train at 5 Hz fMRI pre vs. post rTMS	Somatosensory	Single session no effects in frequency discrimination task in patients linked to decrease in GPi
	5 HC			
Benninger et al., 2011	12 FHD (6 sham)	Prolonged sessions (3 in 1 week) Cathodal tDCS	M1 contralateral to FHD	Prolonged sessions of tDCS yielded no positive effects in clinical measures nor handwriting and cortical excitability
Kimberley et al., 2013	12 FHD	Prolonged session (5 days) at 1 Hz rTMS	Dorsal premotor	Prolonged sessions yielded beneficial effects in pen force at day 1 and 5
Furuya et al., 2014	10 FHC (pianists) 10 HC	Single session of tDCS (cathodal or anodal over affected or unaffected side)	M1	Single session yielded rhythm sequence improvement using cathodal tDCS over affected cortex
Sadnicka et al., 2014	10 WC	Single session anodal tDCS (sham controlled)	Cerebellum	Single session tDCS revealed no positive effects in clinical measures
Koch et al., 2014	18 cervical dystonia	Prolonged sessions (2 weeks) cTBS	Bilateral cerebellum	Prolonged sessions yielded positive acute results (immediate effect after 2-week cTBS) in clinical scales
Bharath et al., 2015	19 WC 20 HC	Single session (1 day) rTMS train at 1 Hz; fMRI pre vs. post	Premotor	Single session reduction in left cerebellum, thalamus, globus pallidus, putamen, bilateral supplementary motor area, medial prefrontal lobe

WC, writer's cramp; HC, healthy controls; FHD, focal hand dystonia; ICD, Idiopathic cervical dystonia; CD, cerebellar dystonia; cTBS, continuous theta burst stimulation; rTMS, repetitive transcranial magnetic stimulation; tACS, transcranial alternating current stimulation; AMT, active motor threshold; tDCS, transcranial direct current stimulation; M1, primary motor cortex; SMA, supplementary motor area.

The apparent efficient parameters to find positive results in dystonia seem to be closely associated to the number of stimulation sessions. Some single session studies have shown effective results (Murase et al., 2005; Tyvaert et al., 2006; Furuya et al., 2014) but are less persistent across time. This single session protocols stimulating premotor regions (at low frequencies) reported motor improvement (hand writing) in focal hand dystonia patients (Siebner et al., 1999; Lefaucheur et al., 2004; Murase et al., 2005). Others reported beneficial clinical effects, as measured by subjective clinical evaluations using 1 Hz rTMS (Murase et al., 2005; Tyvaert et al., 2006), but not always single session turns useful in dystonia (using 5 Hz rTMS; Schneider et al., 2010). Moreover, single sessions are influenced by patient's expectancy or state-dependent effects. Studies that opted for multiple sessions (5 consecutive days) however provide positive and promising results in clinical terms (Lefaucheur et al., 2004; Borich et al., 2009; Angelakis et al., 2013; Kimberley et al., 2013; Koch et al., 2014). Following multiple sessions rTMS, plastic changes in dystonic patients lasted 10 days post-treatment (Borich et al., 2009) and importantly, subjective perception of well-being was maintained for a 3-week period (Havrankova et al., 2010). Others reported only acute amelioration of dystonic signs after cTBS, however after cerebellar stimulation (Koch et al., 2014) thus suggesting premotor regions as responsive for multiple neuromodulation sessions in dystonia.

Regarding rTMS stimulation protocols, the disparity in frequency of stimulation (i.e., low vs. high frequencies) is a solid factor of variability in the current literature. Most of the available literature reports low frequencies (see Table 3), although higher ones, i.e., cTBS, do produce enhanced clinical effects. In Koch et al. (2014), 2-week of TBS applied bilaterally over the cerebellum was compared against a sham TBS condition. Patients under the active stimulation showed ameliorated clinical conditions acutely, but not persistently, with a marked decrease in muscle contraction evaluated by a blinded neurologist. In a similar protocol, following 1 Hz rTMS applied over the left somatosensory parietal region in WC patients (Havrankova et al., 2010), patients showed subjective and objective improvements in writing quality during a 3-week time period. Similarly, using 1 Hz rTMS over dorsal premotor area produced positive results in pen force use and general patients mobility (Kimberley et al., 2013). Such increment in patients response to rTMS may be driven by the fact that low frequency rTMS seems to modulate somatosensory integration in patients with dystonia and WC (Bäumer et al., 2007). Thus, the working hypothesis is that use of repeated sessions may induce cortical plasticity that induces facilitation of sensory outputs or facilitation of contralateral hemisphere (Bharath et al., 2015) to control motor functions. Further evidence is urgently needed to confirm the use of multiple rTMS sessions and to determine ways of prolonging its duration.

**Table 3:** **rTMS application on essential tremor**.

**References**	**Sample**	**TMS protocol**	**Anatomical localization**	**Main findings**
Gironell et al., 2002	10 ET	Single sessions (2 days) Active vs. sham	Cerebellum	Single session acute rTMS beneficial effects on tremor, dissipated in 1 h
		rTMS train at 1 Hz		
Avanzino et al., 2009	15 ET	Single session	Right cerebellum	Single session rTMS yielded beneficial effects on tremor
	11 HC	rTMS train at 1 Hz		
Hellriegel et al., 2012	10 ET	Single sessions (2 days) cTBS 3 pulse bursts at 50 Hz	Left M1	Single session cTBS M1 produced subclinical beneficial effects
	10 HC	80 vs. 30% AMT		
Popa et al., 2013	11 ET	Prolonged sessions (5 consecutive days)	Bilateral cerebellum	Prolonged Session rTMS yielded beneficial effects on tremor during 3 weeks
		rTMS train at 1 Hz		
Chuang et al., 2014	13 ET 18 HC	Single sessions (3 days) cTBS 3 pulse bursts at 50 Hz	M1, premotor and sham	Single session cTBS modulated cortical excitability for shorter duration in ET patients

ET, essential tremor; HC, healthy controls; cTBS, continuous theta burst stimulation; rTMS, repetitive transcranial magnetic stimulation; AMT, active motor threshold; M1, primary motor cortex.

Stimulation over cortical premotor and motor regions connecting with basal ganglia renders a potential treatment in dystonia characterized by functional and compensatory changes in the subcortical regions. Still, greater accuracy in the protocols used to induce subcortical changes are needed. TMS studies trying to induce enhancement of dystonic signs have mostly tackled regions part of the basal ganglia motor loops, i.e., motor, premotor, SMA, and somatosensory regions. The necessity to test alternative TMS protocols under different dystonic symptoms or use stimulation techniques in combination with medication or rehabilitation is obvious.

## Turning down hyperactive cerebello-thalamic loops in essential tremor with rTMS

ET is a hyperkinetic motor disorder that affects one or more body parts by inducing involuntary and rhythmic movements. This may occur in a single limb or at any body part, such as a chin or head with larger prevalence in upper limbs (Helmich et al., 2013). Typically, is presented while moving, bilaterally or kinetic tremor that is visible and persistent. Today, the use of pharmacological in treatment of ET remains poor and unsatisfactory (Louis, 2015). In contrast, surgical treatment is effective in reducing hand tremor in 95% of patients and improved function in 74%, however with added potential risks being an invasive approach (Sandvik et al., 2012).

ET has been associated with altered oscillatory activity in the motor loop involving the cerebello-thalamo-cortical network (Pinto et al., 2003). Several imaging and animal evidence are in keeping with this view of the disease. Indeed, dysfunctional activities (measured as fMRI or PET) and anatomical changes (gray and white matter atrophies) have been found in the well-known *tremor network* (Hallett, 2014), as well as in a plethora of other brain regions involving M1, GPi, thalamus, or cerebellum (Passamonti et al., 2011). However, both functional and structural imaging studies reported convergent findings about the role of the cerebellum as the most consistent area of pathology in ET. This hypothesis has also been confirmed by recent post-mortem studies (Louis et al., 2007, 2011) where it was demonstrated that the average amount of cerebellar Purkinje cells is reduced 25% in tremor patients compared to controls.

Either motor or cerebellum regions are the main target regions to use in neuromodulation for ET. This is mainly guided following results from cortical infarction over motor regions, in which ET motor signs disappeared (Le Pira et al., 2004; Kim et al., 2006). Similarly, single magnetic pulses over M1 seemed to modulate postural tremor in PD patients (Pascual-Leone et al., 1994). These results are in part explained by the correlated frequencies in hand tremor and cortical activity (Hellwig et al., 2001). Yet, rTMS studies trying to apply long-lasting modulatory stimulation in ET have focused over cerebellum and M1 (Gironell et al., 2002, 2014; Avanzino et al., 2009; Hellriegel et al., 2012; Popa et al., 2013; Chuang et al., 2014).

Overall, four studies reported positive anti-tremoric effects using prolonged sessions of rTMS, for a time-period of 3 weeks in ET patients refractory to medical treatment (see Table 3). Beneficial clinical effects assessed by a tremor rating scale (Fahn-Tolosa-Marin tremor scale) were shown as acute and lasting reductions in tremor amplitude and substantial improvement in functional disability (drawing, writing) after rTMS (Popa et al., 2013). Moreover, baseline functional connectivity showed impaired activity in the cerebello-thalamic-cortical loop, a dysfunction that was reset back to near normal levels after rTMS (Popa et al., 2013). The lack of a sham group leaves their results as pending to rule out possible placebo effects.

Historically, the first rTMS application on ET patients was performed by Gironell et al. (2002), who reported acute positive effects after a single session of 1 Hz rTMS over the cerebellum. The study was double blind, crossover, and placebo-controlled design. Their results were significant just in acute evaluation on subjective assessments performed by patients. The nature of their study was exploratory with limited sample size and makes results hard to interpret due to its moderate and transient effects. Next, a second study (Avanzino et al., 2009) using a single session of unilateral 1 Hz cerebellar TMS stimulation, also reported a transient improvement in motor scores evaluated using a tapping task. However, no translational results into clinical scores were found. By contrast, inhibitory cTBS of the left primary motor hand area for 2 consecutive days yielded significant motor benefits by reducing tremor total power, assessed with an accelerometer (Hellriegel et al., 2012). Similarly, Chuang et al. (2014) were interested to alter motor cortical dysfunction in ET by applying cTBS over the primary motor and premotor cortices. They found that cTBS was capable of producing a suppressive effect on motor cortical excitability in ET patients, but the effects lasted for a significantly shorter time compared with the effect produced in healthy individuals. Clinically speaking, tremor amplitude was decreased significantly after cTBS but the tremor frequency remained unchanged. The authors concluded that inhibitory circuits within the motor cortex are aberrant and less modifiable in ET patients.

Mechanistically, cerebellar rTMS seems to turn back-to-normal the altered activity in tremor by re-establishing an appropriate synaptic plasticity involved in programming of motor plans (Ito, 2008), resulting in the most plausible candidate target for ET. It turns, thus, possible to boost tremor sign reduction using rTMS protocols with bilateral cerebellar stimulation and low frequency types under multiple rTMS sessions.

## Conclusions

**Figure 1:**
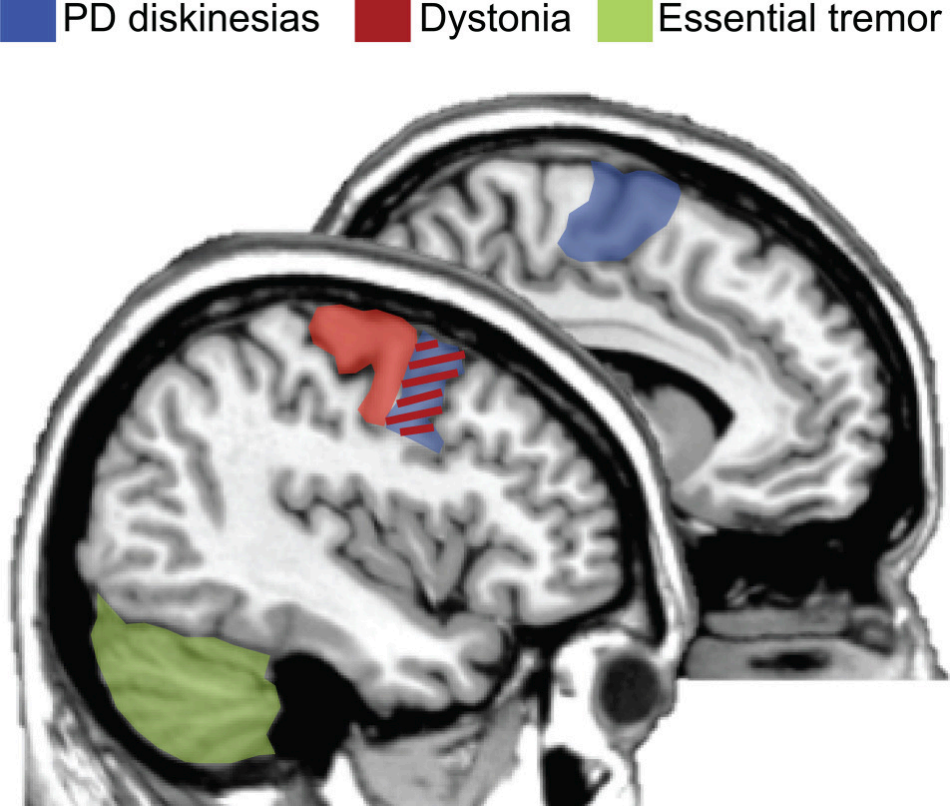
**Optimal brain targets of stimulation for therapeutic purposes**. rTMS targeting the premotor regions and supplementary motor area (SMA, colored in blue) has been demonstrated as the most plausible site of stimulation for reducing hyperkinetic motor disorders in PD patients with levodopa-induced dyskinesias. Moreover, either the premotor or the primary motor cortices (colored in red) are the most frequently used cortical targets for dystonic patients. Finally, based on the literature, the cerebellum (colored in green) has been proposed as the best target for maximizing the effectiveness of rTMS in patients with essential tremor. Of interest, the premotor region is an effective region for two hyperkinetic disorders: dyskinesias and dystonia (colored in red/blue). Figure summarizes (Koch et al., 2005; Brusa et al., 2006; Tyvaert et al., 2006; Popa et al., 2013).

The results of this systematic review show that there was not a clear consensus on optimal protocols to be used for these motor disorders. Future studies are key to consolidate the use of rTMS in this clinical context in order to reduce hyperkinetic brain dysfunctions. However, some positive results give clinical researchers hints of effective neuromodulatory paradigms and uses. Beneficial effects will most likely be boosted if: (i) prolonged sessions are possible, (ii) the use of low frequency rTMS (i.e., 1 Hz), (iii) samples selection restricted to those patients refractory to regular medical treatment, and (iv) choosing the adequate target based on known cortical regions altered in pathophysiological models. Figure 1 represents a summary of candidate regions for treating hyperkinetic dysfunction based on the present literature. Optimal cortical regions that have been demonstrated to maximize the effectiveness of rTMS protocols are: (i) the premotor cortex and SMA for dyskinesias in PD; (ii) the motor and premotor cortices for dystonic patients; and (iii) the cerebellum for patients with ET.

Still, basic procedures or techniques, such as cTBS in tremor or combined therapies (different motor rehabilitation programs and rTMS) have not yet been applied in these disorders. Also, if rTMS protocols are used with patient samples grouped by disease onset and symptom type we may expand knowledge on patient-dependent states and how TMS may modulate differently at each disease stage or symptomatology.

The reader should also note that in this review we neglected some other important hyperkinetic movement disorders such as, Huntington's disease and Tourette syndrome. With respect to Huntington's disease where the application of rTMS for therapeutic purpose is in its relative infancy (Berardelli and Suppa, 2013; Philpott et al., 2013), the large amount of works in psychiatric realm supported the notion that non-invasive brain stimulation is widely recognized as a alternative non-pharmacological approach for decreasing the frequency and intensity of tics in patients with Tourette syndrome (Bloch et al., 2014).

To sum up, the current literature on therapeutic trials of rTMS in hyperkinetic movement disorders patients is still ambiguous, and there is need of well-controlled multicenter randomized clinical trials to define the most effective protocol. However, advancements in technology, as well as, in pathophysiological understanding will improve the effectiveness of this safe and potentially therapeutic option in hyperkinetic movement disorder patients.

## Author contributions

IO designed and wrote the manuscript. AC designed and wrote the manuscript. AQ wrote the manuscript

### Conflict of interest statement

The authors declare that the research was conducted in the absence of any commercial or financial relationships that could be construed as a potential conflict of interest.

## References

[B1] Alexander G. E. DeLong M. R. Strick P. L. (1986). Parallel organization of functionally segregated circuits linking the basal ganglia and cortex. Ann. Rev. Neurosci. 9, 357–381. 10.1146/annurev.ne.09.030186.002041 3085570

[B2] Allam N. Brasil-Neto J. P. Brandão P. Weiler F. Barros Filho J. d. Tomaz C. (2007). Relief of primary cervical dystonia symptoms by low frequency transcranial magnetic stimulation of the premotor cortex: case report. Arq. Neuropsiquiatr. 65, 697–699. 10.1590/S0004-282X2007000400030 17876418

[B3] Angelakis E. Liouta E. Andreadis N. Leonardos A. Ktonas P. Stavrinou L. C.. (2013). Transcranial alternating current stimulation reduces symptoms in intractable idiopathic cervical dystonia: a case study. Neurosci. Lett. 533, 39–43. 10.1016/j.neulet.2012.11.007 23149130

[B4] Avanzino L. Bove M. Tacchino A. Ruggeri P. Giannini A. Trompetto C.. (2009). Cerebellar involvement in timing accuracy of rhythmic finger movements in essential tremor. Eur. J. Neurosci. 30, 1971–1979. 10.1111/j.1460-9568.2009.06984.x 19912337

[B5] Bäumer T. Demiralay C. Hidding U. Bikmullina R. Helmich R. C. Wunderlich S.. (2007). Abnormal plasticity of the sensorimotor cortex to slow repetitive transcranial magnetic stimulation in patients with writer's cramp. Mov. Disord. 22, 81–90. 10.1002/mds.21219 17089385

[B6] Bäumer T. Lange R. Liepert J. Weiller C. Siebner H. R. Rothwell J. C.. (2003). Repeated premotor rTMS leads to cumulative plastic changes of motor cortex excitability in humans. Neuroimage 20, 550–560. 10.1016/S1053-8119(03)00310-0 14527615

[B7] Benninger D. H. Lomarev M. Lopez G. Pal N. Luckenbaugh D. A. Hallett M. (2011). Transcranial direct current stimulation for the treatment of focal hand dystonia. Mov. Disord. 26, 1698–1702. 10.1002/mds.23691 21495074 PMC4180819

[B8] Berardelli A. Rothwell J. C. Hallett M. Thompson P. D. Manfredi M. Marsden C. D. (1998). The pathophysiology of primary dystonia. Brain 121(Pt 7), 1195–1212. 10.1093/brain/121.7.1195 9679773

[B9] Berardelli A. Suppa A. (2013). Noninvasive brain stimulation in Huntington's disease. Handb. Clin. Neurol. 116, 555–560. 10.1016/B978-0-444-53497-2.00044-9 24112923

[B10] Berman B. D. Hallett M. Herscovitch P. Simonyan K. (2013). Striatal dopaminergic dysfunction at rest and during task performance in writer's cramp. Brain 136, 3645–3658. 10.1093/brain/awt282 24148273 PMC3859223

[B11] Bharath R. D. Biswal B. B. Bhaskar M. V. Gohel S. Jhunjhunwala K. Panda R.. (2015). Repetitive transcranial magnetic stimulation induced modulations of resting state motor connectivity in writer's cramp. Eur. J. Neurol. 22, 796–805; e53–e54. 10.1111/ene.12653 25623591

[B12] Bloch Y. Arad S. Levkovitz Y. (2014). Deep TMS add-on treatment for intractable tourette syndrome: a feasibility study. World J. Biol. Psychiatry 24, 1–5. 10.3109/15622975.2014.964767 25342253

[B13] Borich M. Arora S. Kimberley T. J. (2009). Lasting effects of repeated rTMS application in focal hand dystonia. Restor. Neurol. Neurosci. 27, 55–65. 10.3233/RNN-2009-0461 19164853 PMC4456689

[B14] Bragg D. C. Armata I. A. Nery F. C. Breakefield X. O. Sharma N. (2011). Molecular pathways in dystonia. Neurobiol. Dis. 42, 136–147. 10.1016/j.nbd.2010.11.015 21134457 PMC3073693

[B15] Breakefield X. O. Blood A. J. Li Y. Hallett M. Hanson P. I. Standaert D. G. (2008). The pathophysiological basis of dystonias. Nat. Rev. Neurosci. 9, 222–234. 10.1038/nrn2337 18285800

[B16] Brighina F. Romano M. Giglia G. Saia V. Puma A. Giglia F.. (2009). Effects of cerebellar TMS on motor cortex of patients with focal dystonia: a preliminary report. Exp. Brain Res. 192, 651–656. 10.1007/s00221-008-1572-9 18815775

[B17] Brooks D. J. Piccini P. Turjanski N. Samuel M. (2000). Neuroimaging of dyskinesia. Ann. Neurol. 47, S154–S158. discussion: S158–S159. 10762143

[B18] Brusa L. Versace V. Koch G. Iani C. Stanzione P. Bernardi G.. (2006). Low frequency rTMS of the SMA transiently ameliorates peak-dose LID in Parkinson's disease. Clin. Neurophysiol. 117, 1917–1921. 10.1016/j.clinph.2006.03.033 16887383

[B19] Butterworth S. Francis S. Kelly E. McGlone F. Bowtell R. Sawle G. V. (2003). Abnormal cortical sensory activation in dystonia: an fMRI study. Mov. Disord. 18, 673–682. 10.1002/mds.10416 12784271

[B20] Cerasa A. Fasano A. Morgante F. Koch G. Quattrone A. (2014). Maladaptive plasticity in levodopa-induced dyskinesias and tardive dyskinesias: old and new insights on the effects of dopamine receptor pharmacology. Front. Neurol. 5:49. 10.3389/fneur.2014.00049 24782822 PMC3988357

[B21] Cerasa A. Koch G. Donzuso G. Mangone G. Morelli M. Brusa L.. (2015). A network centred on the inferior frontal cortex is critically involved in levodopa-induced dyskinesias. Brain 138, 414–427. 10.1093/brain/awu329 25414038

[B22] Cerasa A. Messina D. Pugliese P. Morelli M. Lanza P. Salsone M.. (2011). Increased prefrontal volume in PD with levodopa-induced dyskinesias: a voxel-based morphometry study. Mov. Disord. 26, 807–812. 10.1002/mds.23660 21384430

[B23] Cerasa A. Morelli M. Augimeri A. Salsone M. Novellino F. Gioia M. C.. (2013a). Prefrontal thickening in PD with levodopa-induced dyskinesias: new evidence from cortical thickness measurement. Parkinsonism Relat. Disord. 19, 123–125. 10.1016/j.parkreldis.2012.06.003 22742954

[B24] Cerasa A. Pugliese P. Messina D. Morelli M. Gioia M. C. Salsone M.. (2012). Prefrontal alterations in Parkinson's disease with levodopa-induced dyskinesia during fMRI motor task. Mov. Disord. 27, 364–371. 10.1002/mds.24017 22076870

[B25] Cerasa A. Quattrone A. (2014a). May hyperdirect pathway be a plausible neural substrate for understanding the rTMS-related effects on PD patients with levodopa-induced dyskinesias? Brain Stimul. 7, 488–489. 10.1016/j.brs.2014.01.007 24507575

[B26] Cerasa A. Quattrone A. (2014b). May stimulation of the pre-SMA become a new therapeutic target for PD patients with levodopa-induced dyskinesias? Brain Stimul. 7, 335–336. 10.1016/j.brs.2013.12.003 24389502

[B27] Cerasa A. Salsone M. Morelli M. Pugliese P. Arabia G. Gioia C. M.. (2013b). Age at onset influences neurodegenerative processes underlying PD with levodopa-induced dyskinesias. Parkinsonism Relat. Disord. 19, 883–888. 10.1016/j.parkreldis.2013.05.015 23769805

[B28] Chuang W.-L. Huang Y.-Z. Lu C.-S. Chen R.-S. (2014). Reduced cortical plasticity and GABAergic modulation in essential tremor. Mov. Disord. 29, 501–507. 10.1002/mds.25809 24449142

[B29] Detante O. Vercueil L. Thobois S. Broussolle E. Costes N. Lavenne F.. (2004). Globus pallidus internus stimulation in primary generalized dystonia: a H215O PET study. Brain 127, 1899–1908. 10.1093/brain/awh213 15231585

[B30] Diamond A. Jankovic J. (2005). The effect of deep brain stimulation on quality of life in movement disorders. J. Neurol. Neurosurg. Psychiatry 76, 1188–1193. 10.1136/jnnp.2005.065334 16107348 PMC1739801

[B31] Draganski B. Thun-Hohenstein C. Bogdahn U. Winkler J. May A. (2003). “Motor circuit” gray matter changes in idiopathic cervical dystonia. Neurology 61, 1228–1231. 10.1212/01.WNL.0000094240.93745.83 14610125

[B32] Edwards M. J. Talelli P. Rothwell J. C. (2008). Clinical applications of transcranial magnetic stimulation in patients with movement disorders. Lancet Neurol. 7, 827–840. 10.1016/S1474-4422(08)70190-X 18703005

[B33] Eidelberg D. Moeller J. R. Ishikawa T. Dhawan V. Spetsieris P. Przedborski S.. (1995). The metabolic topography of idiopathic torsion dystonia. Brain 118(Pt 6), 1473–1484. 10.1093/brain/118.6.1473 8595478

[B34] Elahi B. Elahi B. Chen R. (2009). Effect of transcranial magnetic stimulation on Parkinson motor function–systematic review of controlled clinical trials. Mov. Disord. 24, 357–363. 10.1002/mds.22364 18972549

[B35] Fasano A. Lozano A. M. (2015). Deep brain stimulation for movement disorders: 2015 and beyond. Curr. Opin. Neurol. 28, 423–436. 10.1097/WCO.0000000000000226 26110808

[B36] Filipović S. R. Bhatia K. P. Rothwell J. C. (2013). 1-Hz repetitive transcranial magnetic stimulation and diphasic dyskinesia in Parkinson's disease. Mov. Disord. 28, 245–246. 10.1002/mds.25261 23144006

[B37] Filipovic S. R. Rothwell J. C. van de Warrenburg B. P. Bhatia K. (2009). Repetitive transcranial magnetic stimulation for levodopa-induced dyskinesias in Parkinson's disease. Mov. Disord. 24, 246–253. 10.1002/mds.22348 18951540

[B38] Furuya S. Nitsche M. A. Paulus W. Altenmüller E. (2014). Surmounting retraining limits in Musicians' dystonia by transcranial stimulation. Ann. Neurol. 75, 700–707. 10.1002/ana.24151 24706370

[B39] George M. S. Taylor J. J. Short E. B. (2013). The expanding evidence base for rTMS treatment of depression. Curr. Opin. Psychiatry 26, 13–18. 10.1097/YCO.0b013e32835ab46d 23154644 PMC4214363

[B40] Gironell A. Aguilar S. Torres V. Pagonabarraga J. (2014). Transcranial direct current stimulation of the cerebellum in essential tremor?: a controlled study deep transcranial magnetic stimulation in a woman with chronic tinnitus?: clinical and fMRI findings. seeking relief from a symptom and finding vivid memor. BRS 7, 491–492. 10.1016/j.brs.2014.02.001 24582371

[B41] Gironell A. Kulisevsky J. Lorenzo J. Barbanoj M. Pascual-Sedano B. Otermin P. (2002). Transcranial magnetic stimulation of the cerebellum in essential tremor: a controlled study. Arch. Neurol. 59, 413–417. 10.1001/archneur.59.3.413 11890845

[B42] Hallett M. (2014). Tremor: pathophysiology. Parkinsonism Relat. Disord. 20(Suppl. 1), S118–S122. 10.1016/S1353-8020(13)70029-4 24262161

[B43] Hamani C. Saint-Cyr J. A. Fraser J. Kaplitt M. Lozano A. M. (2004). The subthalamic nucleus in the context of movement disorders. Brain 127, 4–20. 10.1093/brain/awh029 14607789

[B44] Havrankova P. Jech R. Walker N. D. Operto G. Tauchmanova J. Vymazal J.. (2010). Repetitive TMS of the somatosensory cortex improves writer's cramp and enhances cortical activity. Neuro Endocrinol. Lett. 31, 73–86. 20150883

[B45] Hellriegel H. Schulz E. M. Siebner H. R. Deuschl G. Raethjen J. H. (2012). Continuous theta-burst stimulation of the primary motor cortex in essential tremor. Clin. Neurophysiol. 123, 1010–1015. 10.1016/j.clinph.2011.08.033 21982298

[B46] Hellwig B. Häussler S. Schelter B. Lauk M. Guschlbauer B. Timmer J.. (2001). Tremor-correlated cortical activity in essential tremor. Lancet 357, 519–523. 10.1016/S0140-6736(00)04044-7 11229671

[B47] Helmich R. C. Toni I. Deuschl G. Bloem B. R. (2013). The pathophysiology of essential tremor and parkinson's tremor. Curr. Neurol. Neurosci. Rep. 13:378. 10.1007/s11910-013-0378-8 23893097

[B48] Herz D. M. Haagensen B. N. Christensen M. S. Madsen K. H. Rowe J. B. Løkkegaard A.. (2014). The acute brain response to levodopa heralds dyskinesias in Parkinson disease. Ann. Neurol. 75, 829–836. 10.1002/ana.24138 24889498 PMC4112717

[B49] Herz D. M. Haagensen B. N. Christensen M. S. Madsen K. H. Rowe J. B. Løkkegaard A.. (2015). Abnormal dopaminergic modulation of striato-cortical networks underlies levodopa-induced dyskinesias in humans. Brain 138, 1658–1666. 10.1093/brain/awv096 25882651 PMC4614130

[B50] Huang Y. Z. Edwards M. J. Rounis E. Bhatia K. P. Rothwell J. C. (2005). Theta burst stimulation of the human motor cortex. Neuron 45, 201–206. 10.1016/j.neuron.2004.12.033 15664172

[B51] Huang Y. Z. Rothwell J. C. Lu C. S. Wang J. Chen R. S. (2011). Restoration of motor inhibition through an abnormal premotor-motor connection in dystonia. Mov. Disord. 25, 696–703. 10.1002/mds.22814 20309999 PMC3000921

[B52] Iacono D. Geraci-Erck M. Peng H. Rabin M. L. Kurlan R. (2015). Reduced number of pigmented neurons in the substantia nigra of dystonia patients? Findings from extensive neuropathologic, immunohistochemistry, and quantitative analyses. Tremor Other Hyperkinet. Mov. (N.Y). 5:tre-5-301. 10.7916/D8T72G9G 26069855 PMC4458735

[B53] Ibáñez V. Sadato N. Karp B. Deiber M. P. Hallett M. (1999). Deficient activation of the motor cortical network in patients with writer's cramp. Neurology 53, 96–105. 10408543 10.1212/wnl.53.1.96

[B54] Ito M. (2008). Control of mental activities by internal models in the cerebellum. Nat. Rev. Neurosci. 9, 304–313. 10.1038/nrn2332 18319727

[B55] Kehagia A. A. Barker R. A. Robbins T. W. (2013). Cognitive impairment in Parkinson's disease: the dual syndrome hypothesis. Neurodegener. Dis. 11, 79–92. 10.1159/000341998 23038420 PMC5079071

[B56] Kim J.-S. Park J.-W. Kim W.-J. Kim H.-T. Kim Y.-I. Lee K.-S. (2006). Disappearance of essential tremor after frontal cortical infarct. Mov. Disord. 21, 1284–1285. 10.1002/mds.20894 16637038

[B57] Kimberley T. J. Borich M. R. Arora S. Siebner H. R. (2013). Multiple sessions of low-frequency repetitive transcranial magnetic stimulation in focal hand dystonia: Clinical and physiological effects. Restor. Neurol. Neurosci. 31, 533–542. 10.3233/RNN-120259 23340117 PMC5149409

[B58] Koch G. (2013). Do studies on cortical plasticity provide a rationale for using non-invasive brain stimulation as a treatment for Parkinson's disease patients? Front. Neurol. 4:180. 10.3389/fneur.2013.00180 24223573 PMC3818583

[B59] Koch G. Brusa L. Caltagirone C. Peppe A. Oliveri M. Stanzione P.. (2005). rTMS of supplementary motor area modulates therapy-induced dyskinesias in Parkinson disease. Neurology 65, 623–625. 10.1212/01.wnl.0000172861.36430.95 16116131

[B60] Koch G. Brusa L. Carrillo F. Lo Gerfo E. Torriero S. Oliveri M.. (2009). Cerebellar magnetic stimulation decreases levodopa-induced dyskinesias in Parkinson disease. Neurology 73, 113–119. 10.1212/WNL.0b013e3181ad5387 19597133

[B61] Koch G. Porcacchia P. Ponzo V. Carrillo F. Cáceres-Redondo M. T. Brusa L.. (2014). Effects of two weeks of cerebellar theta burst stimulation in cervical dystonia patients. Brain Stimul. 7, 564–572. 10.1016/j.brs.2014.05.002 24881805

[B62] Kodama M. Kasahara T. Hyodo M. Aono K. Sugaya M. Koyama Y.. (2011). Effect of low-frequency repetitive transcranial magnetic stimulation combined with physical therapy on L-dopa-induced painful off-period dystonia in Parkinson's disease. Am. J. Phys. Med. Rehabil. 90, 150–155. 10.1097/PHM.0b013e3181fc7ccd 20975525

[B63] Le Pira F. Giuffrida S. Panetta M. R. Lo Bartolo M. L. Politi G. (2004). Selective disappearance of essential tremor after ischaemic stroke. Eur. J. Neurol. 11, 422–423. 10.1111/j.1468-1331.2004.00824.x 15171740

[B64] Lefaucheur J. P. Drouot X. Von Raison F. Ménard-Lefaucheur I. Cesaro P. Nguyen J. P. (2004). Improvement of motor performance and modulation of cortical excitability by repetitive transcranial magnetic stimulation of the motor cortex in Parkinson's disease. Clin. Neurophysiol. 115, 2530–2541. 10.1016/j.clinph.2004.05.025 15465443

[B65] Lehéricy S. Tijssen M. A. J. Vidailhet M. Kaji R. Meunier S. (2013). The anatomical basis of dystonia: current view using neuroimaging. Mov. Disord. 28, 944–957. 10.1002/mds.25527 23893451

[B66] Lerner A. Shill H. Hanakawa T. Bushara K. Goldfine A. Hallett M. (2004). Regional cerebral blood flow correlates of the severity of writer's cramp symptoms. Neuroimage 21, 904–913. 10.1016/j.neuroimage.2003.10.019 15006657

[B67] Louis E. D. (2015). Medication non-adherence in essential tremor. Parkinsonism Relat. Disord. 21, 138–141. 10.1016/j.parkreldis.2014.12.001 25523964 PMC4306620

[B68] Louis E. D. Asabere N. Agnew A. Moskowitz C. B. Lawton A. Cortes E.. (2011). Rest tremor in advanced essential tremor: a post-mortem study of nine cases. J. Neurol. Neurosurg. Psychiatry 82, 261–265. 10.1136/jnnp.2010.215681 20802027

[B69] Louis E. D. Faust P. L. Vonsattel J.-P. G. Honig L. S. Rajput A. Robinson C. A.. (2007). Neuropathological changes in essential tremor: 33 cases compared with 21 controls. Brain 130, 3297–3307. 10.1093/brain/awm266 18025031

[B70] Marsden C. D. (1976). Dystonia: the spectrum of the disease. Res. Publ. Assoc. Res. Nerv. Ment. Dis. 55, 351–367. 1005905

[B71] Marsden C. D. Obeso J. A. Zarranz J. J. Lang A. E. (1985). The anatomical basis of symptomatic hemidystonia. Brain 108(Pt 2), 463–483. 10.1093/brain/108.2.463 4005532

[B72] Middleton F. A. Strick P. L. (2000). Basal ganglia output and cognition: evidence from anatomical, behavioral, and clinical studies. Brain Cogn. 42, 183–200. 10.1006/brcg.1999.1099 10744919

[B73] Murase N. Rothwell J. C. Kaji R. Urushihara R. Nakamura K. Murayama N.. (2005). Subthreshold low-frequency repetitive transcranial magnetic stimulation over the premotor cortex modulates writer's cramp. Brain 128, 104–115. 10.1093/brain/awh315 15483042

[B74] Neumann W.-J. Jha A. Bock A. Huebl J. Horn A. Schneider G.-H.. (2015). Cortico-pallidal oscillatory connectivity in patients with dystonia. Brain 138, 1894–1906. 10.1093/brain/awv109 25935723

[B75] Obeso I. Cho S. S. Antonelli F. Houle S. Jahanshahi M. Ko J. H.. (2013). Stimulation of the pre-SMA influences cerebral blood flow in frontal areas involved with inhibitory control of action. Brain Stimul. 6, 769–776. 10.1016/j.brs.2013.02.002 23545472

[B76] Obeso I. Strafella A. P. (2014a). Boosting response inhibition neural network with rTMS may improve dyskinesias in Parkinson's disease. Brain Stimul. 7, 336–337. 10.1016/j.brs.2013.12.004 24406171

[B77] Obeso I. Strafella A. P. (2014b). Top-down control of dyskinesias in PD using brain stimulation. Brain Stimul. 7, 488. 10.1016/j.brs.2014.01.005 24507577

[B78] Obeso J. A. Rodriguez-Oroz M. C. Rodriguez M. DeLong M. R. Olanow C. W. (2000). Pathophysiology of levodopa-induced dyskinesias in Parkinson's disease: problems with the current model. Ann. Neurol. 47(4 Suppl.), S22–S32. discussion: S32-S34. 10762129

[B79] Odergren T. Stone-Elander S. Ingvar M. (1998). Cerebral and cerebellar activation in correlation to the action-induced dystonia in writer's cramp. Mov. Disord. 13, 497–508. 10.1002/mds.870130321 9613744

[B80] Oga T. Honda M. Toma K. Murase N. Okada T. Hanakawa T.. (2002). Abnormal cortical mechanisms of voluntary muscle relaxation in patients with writer's cramp: an fMRI study. Brain 125, 895–903. 10.1093/brain/awf083 11912121

[B81] Otsuka M. Ichiya Y. Shima F. Kuwabara Y. Sasaki M. Fukumura T.. (1992). Increased striatal 18F-dopa uptake and normal glucose metabolism in idiopathic dystonia syndrome. J. Neurol. Sci. 111, 195–199. 10.1016/0022-510X(92)90068-V 1431986

[B82] Pascual-Leone A. Valls-Solé J. Toro C. Wassermann E. M. Hallett M. (1994). Resetting of essential tremor and postural tremor in Parkinson's disease with transcranial magnetic stimulation. Muscle Nerve 17, 800–807. 10.1002/mus.880170716 8008009

[B83] Passamonti L. Novellino F. Cerasa A. Chiriaco C. Rocca F. Matina M. S.. (2011). Altered cortical-cerebellar circuits during verbal working memory in essential tremor. Brain 134, 2274–2286. 10.1093/brain/awr164 21747127

[B84] Philpott A. L. Fitzgerald P. B. Cummins T. D. Georgiou-Karistianis N. (2013). Transcranial magnetic stimulation as a tool for understanding neurophysiology in Huntington's disease: a review. Neurosci. Biobehav. Rev. 37, 1420–1433. 10.1016/j.neubiorev.2013.05.009 23727400

[B85] Picazio S. Koch G. (2015). Is motor inhibition mediated by cerebello-cortical interactions? Cerebellum 14, 47–49. 10.1007/s12311-014-0609-9 25283181

[B86] Pinto A. D. Lang A. E. Chen R. (2003). The cerebellothalamocortical pathway in essential tremor. Neurology 60, 1985–1987. 10.1212/01.WNL.0000065890.75790.29 12821747

[B87] Popa T. Russo M. Vidailhet M. Roze E. Lehéricy S. (2013). Brain Stimulation Cerebellar rTMS stimulation may induce prolonged clinical bene fi ts in essential tremor, and subjacent changes in functional connectivity?: an open label trial. Brain Stimul. 6, 175–179. 10.1016/j.brs.2012.04.009 22609238

[B88] Pujol J. Roset-Llobet J. Rosinés-Cubells D. Deus J. Narberhaus B. Valls-Solé J.. (2000). Brain cortical activation during guitar-induced hand dystonia studied by functional MRI. Neuroimage 12, 257–267. 10.1006/nimg.2000.0615 10944408

[B89] Rascol O. Sabatini U. Brefel C. Fabre N. Rai S. Senard J. M.. (1998). Cortical motor overactivation in parkinsonian patients with L-dopa-induced peak-dose dyskinesia. Brain 121(Pt 3), 527–533. 10.1093/brain/121.3.527 9549528

[B90] Ridding M. C. Rothwell J. C. (2007). Is there a future for therapeutic use of transcranial magnetic stimulation? Nat. Rev. Neurosci. 8, 559–567. 10.1038/nrn2169 17565358

[B91] Rothwell J. C. Obeso J. A. (2015). Can levodopa-induced dyskinesias go beyond the motor circuit? Brain 138, 242–244. 10.1093/brain/awu365 25627235

[B92] Sadnicka A. Hamada M. Bhatia K. P. Rothwell J. C. Edwards M. J. (2014). Cerebellar stimulation fails to modulate motor cortex plasticity in writing dystonia. Mov. Disord. 29, 1304–1307. 10.1002/mds.25881 24797030

[B93] Sandvik U. Koskinen L.-O. Lundquist A. Blomstedt P. (2012). Thalamic and subthalamic deep brain stimulation for essential tremor: where is the optimal target? Neurosurgery 70, 840–5. discussion: 845–846. 10.1227/NEU.0b013e318236a809 22426044

[B94] Schneider S. A. Pleger B. Draganski B. Cordivari C. Rothwell J. C. Bhatia K. P.. (2010). Modulatory effects of 5Hz rTMS over the primary somatosensory cortex in focal dystonia - An fMRI-TMS Study. Mov. Disord. 25, 76–83. 10.1002/mds.22825 20058321 PMC2929458

[B95] Siebner H. R. Tormos J. M. Ceballos-Baumann A. O. Auer C. Catala M. D. Conrad B.. (1999). Low-frequency repetitive transcranial magnetic stimulation of the motor cortex in writer's cramp. Neurology 52, 529–537. 10.1212/WNL.52.3.529 10025782

[B96] Tyvaert L. Houdayer E. Devanne H. Monaca C. Cassim F. Derambure P. (2006). The effect of repetitive transcranial magnetic stimulation on dystonia: a clinical and pathophysiological approach. Neurophysiol. Clin. 36, 135–143. 10.1016/j.neucli.2006.08.007 17046608

[B97] Vernon A. C. Modo M. (2012). Do levodopa treatments modify the morphology of the parkinsonian brain? Mov. Disord. 27, 166–167. 10.1002/mds.24018 22076912

[B98] Vidailhet M. Vercueil L. Houeto J.-L. Krystkowiak P. Benabid A.-L. Cornu P.. (2005). Bilateral deep-brain stimulation of the globus pallidus in primary generalized dystonia. N. Engl. J. Med. 352, 459–467. 10.1056/NEJMoa042187 15689584

[B99] Vitek J. L. Chockkan V. Zhang J. Y. Kaneoke Y. Evatt M. DeLong M. R.. (1999). Neuronal activity in the basal ganglia in patients with generalized dystonia and hemiballismus. Ann. Neurol. 46, 22–35. 10401777 10.1002/1531-8249(199907)46:1<22::aid-ana6>3.0.co;2-z

[B100] Wagle-Shukla A. Angel M. J. Zadikoff C. Enjati M. Gunraj C. Lang A. E.. (2007). Low-frequency repetitive transcranial magnetic stimulation for treatment of levodopa-induced dyskinesias. Neurology 68, 704–705. 10.1212/01.wnl.0000256036.20927.a5 17325284

[B101] Wu T. Hallett M. (2013). Reply: the cerebellum in Parkinson's disease and parkinsonism in cerebellar disorders. Brain 136:e249. 10.1093/brain/aws360 23739172

[B102] Zandbelt B. B. Bloemendaal M. Hoogendam J. M. Kahn R. S. Vink M. (2013). Transcranial magnetic stimulation and functional MRI reveal cortical and subcortical interactions during stop-signal response inhibition. J. Cogn. Neurosci. 25, 157–174. 10.1162/jocn_a_00309 23066733

[B103] Zheng Z. Pan P. Wang W. Shang H. (2012). Neural network of primary focal dystonia by an anatomic likelihood estimation meta-analysis of gray matter abnormalities. J. Neurol. Sci. 316, 51–55. 10.1016/j.jns.2012.01.032 22349356

[B104] Zhuang P. Li Y. Hallett M. (2004). Neuronal activity in the basal ganglia and thalamus in patients with dystonia. Clin. Neurophysiol. 115, 2542–2557. 10.1016/j.clinph.2004.06.006 15465444

